# Relationship Between Use of Dementia-Specific Nursing Institutions and Psychotropic Drugs, Mortality, and Morbidity

**DOI:** 10.1192/bjo.2024.487

**Published:** 2024-08-01

**Authors:** Jung Suk Lee, Byung Ook Lee

**Affiliations:** NHIS Ilsan Hospital, Goynag, Korea, Republic of

## Abstract

**Aims:**

In South Korea, to care for patients with dementia, a new dementia-specific nursing institution has been established that, unlike general nursing institutions, uses shared living rooms and provides customized programs for dementia. This study aims to investigate the effectiveness of dementia-specific nursing institutions. For this purpose, whether psychotropic drugs (antipsychotics, antidepressants, sedatives, mood stabilizers) used to treat behavioral and psychological symptoms of dementia (BPSD) are prescribed, and the mortality and morbidity rates mentioned as side effects of psychotropic drugs (cerebrovascular disease, fall-related fractures, pneumonia, pressure ulcers) varied depending on the use of a dementia-specific nursing institution.

**Methods:**

Using the National Health Insurance Service's customized and Long-Term Care Insurance databases for older people, we collected data over the four years since the introduction of dementia-specific nursing institutions. Among patients with dementia aged 65 years or older, those who used dementia-specific nursing institutions and those who used general nursing institutions were matched for gender, age, history of cerebrovascular disease, disability, comorbidities, and history of taking psychotropic drugs. Thus, 835 users of dementia-specific nursing institutions and 2,505 users of general nursing institutions were analyzed. During the study period, the subjects' use of psychotropic drugs, mortality, and morbidity (cerebrovascular disease, fall-related fractures, pneumonia, and pressure ulcers) were determined. After controlling for variables such as Activities of Daily Living (ADL) scores, the effect of using a dementia-specific nursing institution on mortality and morbidity was analyzed using a logistic regression model.

**Results:**

Users of dementia-specific nursing institutions were more likely to be prescribed antipsychotics, antidepressants, and sedatives during the study period compared with users of general nursing institutions. Also, users of dementia-specific nursing institutions had a lower mortality rate and lower morbidity rates of pneumonia and pressure ulcers than users of general nursing institutions.

**Conclusion:**

Users of dementia-specific nursing institutions had significantly lower mortality rates and morbidity rates of pneumonia and pressure ulcers. This is attributed to dementia-specific nursing institutions encouraging social interaction and physical activity by providing shared living rooms and specialized programs catered towards patients with dementia. However, since the influence of other confounding variables cannot be ruled out, more precisely designed research is needed in the future.

